# Expression of both poly r(C) binding protein 1 (PCBP1) and miRNA-3978 is suppressed in peritoneal gastric cancer metastasis

**DOI:** 10.1038/s41598-017-15448-9

**Published:** 2017-11-14

**Authors:** Fu-jian Ji, Yuan-yu Wu, Zhe An, Xue-song Liu, Jun-nan Jiang, Fang-fang Chen, Xue-dong Fang

**Affiliations:** 1 Department of Gastrointestinal Colorectal and Anal Surgery, China-Japan Union Hospital of Jilin University, Changchun, 130033 China; 2 Department of Cardiology, China-Japan Union Hospital of Jilin University, Changchun, 130033 China

## Abstract

The expression of legumain which has been shown overexpressed in patients with metastatic gastric cancer is positively correlated to both disease progression and outcome, and negatively correlated to microRNA (miR)−3978 expression. The RNA-binding protein, poly r(C) binding protein 1 (PCBP1) was the most downregulated protein in the metastatic tissue specimens. Quantitative real-time PCR showed that *PCBP1* expression is transcriptionally downregulated in peritoneal metastasis tissues. RNA immunoprecipitation experiments showed that PCBP1 and miR-3978 are sequestered in normal peritoneal tissue, but the complex is disrupted following metastatic progression. *PCBP1* expression mimicked miR-3978 expression across gastric cancer patients. Finally, replenishment of *PCBP1* or miR-3978 expression in the peritoneal metastasis cell line MKN45 decreased legumain protein expression and chemosensitized the cells to treatment with docetaxel. However, replenishment of one and concomitant depletion of the other failed to induce chemosensitivity to docetaxel. Replenishment of miR-3978 also resulted in induction of PCBP1 protein expression, potentially indicating that miR-3978 expression might downregulate a negative regulator targeting *PCBP1*. Our current study reveals *PCBP1* as an additional biomarker in peritoneal metastasis. *PCBP1* and miR-3978 expression were correlated and suggests a potential interplay of differential miRNA biogenesis and RNA binding protein during metastatic progression.

## Introduction

Approximately 300,000 patients with gastric cancer are projected to die annually in China^1^, 50% of which is due to peritoneal metastasis^2^. Even though radical resection is the accepted mode of treatment in these patients, peritoneal metastasis remains the single largest cause of mortality in patients with gastric cancer^3–6^. Even though most patients already have regional or distant metastasis in patients with gastric cancer, radical resection without knowledge of potent diagnostic and prognostic markers is not highly efficient in preventing progression to peritoneal metastasis^7,8^.

Legumain or asparaginyl endopeptidase (AEP), a member of the lysosomal cysteine endopeptidase, has been found to be overexpressed in patients with metastatic gastric cancer^9–12^. Legumain potentiates metastatic progression by augmenting epithelial to mesenchymal transition by activation of MAPK and Akt signaling pathways^13^. In addition, proteolytic activation of other zymogens mediated by legumain is also a pathway leading to both tumor development and progression *in vivo*^14–16^.

We have earlier shown that microRNA-3978 (miR-3978) regulates legumain expression in normal peritoneum. In gastric cancer patients with peritoneal metastasis legumain expression is induced because miR-3978 expression is suppressed^17^. MiRNAs are evolutionarily conserved 21–23 nucleotides RNAs that regulate post-transcriptional gene expression either by blocking translation or degrading target messenger RNAs (mRNAs) and have been increasingly shown to function as tumor suppressors or oncogenes ^18,19^. MiRNAs can function in both normal and transformed cells and have even been shown to play a role in metastasis^20–23^.

Not much is known about role of miR-3978 in gastric cancer or in normal physiology. There has been one report that suggested differential expression of miR-3978 in lung cancer patients^24^. Given that legumain has been shown to be overexpressed in different metastatic cancers^13–16,25–27^, it is imperative to define precise mechanism that regulates miR-3978 expression in gastric cancer patients with peritoneal metastasis. Our findings in the current study cumulatively indicate that the RNA binding protein, poly r(C) binding protein 1 (PCBP1) or heterogeneous nuclear ribonucleoprotein E1 (hnRNPE1) expression correlates withmiR-3978 expression and that hnRNP E1 itself functions as a suppressor of tumor progression.

## Materials and Methods

### Patient samples

The study protocol was approved by the Institutional Review Board of the China-Japan Union Hospital of Jilin University and all experiments were performed in accordance with relevant guidelines and regulations. A total of 20 patients (12 men and 8 women) who had surgery for gastric cancer between 2014 and 2015 at the China-Japan Union Hospital of Jilin University were recruited in this study. Signed informed consent was obtained from all patients before enrolling for the current study. The mean age of the patients was 61.34 years (range, 39–78 years). The inclusion criteria were presence of peritoneal metastasis at the time of initial presentation as confirmed by two independent pathologists. Paired tumor tissue and normal gastric tissue specimens were obtained from all patients included in the study.

### Western blotting

Cells were lysed in buffer containing 25 mM Tris-HCl pH 7.4, 150 mM NaCl, 1 mM EDTA, 1% NP-40 and 5% glycerol supplemented with complete, Mini protease inhibitor cocktail (Roche Diagnostics, Shanghai, China). Whole cell lysate was resolved on SDS-PAGE and probed with anti-PCBP1 antibody (clone E-2, sc-137249; Santa Cruz Biotechnology, USA) or anti-Legumain antibody^17^. All blots were subsequently stripped, and re-probed with GAPDH (ThermoFisher Scientific, Shanghai, China) to confirm equal loading.

### Mass spectrometry analysis

Tissue (tumor and adjacent normal) obtained from 5 patients were washed thrice with ice-cold PBS before being lysed using NET buffer (50 mmol/L Tris (pH, 7.4), 150 mmol/L NaCl, 0.1% NP40, 1 mmol/L EDTA, 0.25% gelatin, 0.02% sodium azide) supplemented with protease and phosphatase inhibitor cocktail (ThermoFisher Scientific, Shanghai, China). Lysates were centrifuged at 10,000 rcf for 20 minutes at 4 °C and the supernatant were stored at −80 °C until further use. The lysates were dialyzed against PBS before being reduced and alkylated using 5 mM Tris 2-carboxyethyl phosphine (TCEP) and 10 mM iodoacetamide, respectively. Peptides were desalted, lyophilized, before being re-dissolved in 20 µl of HPLC-grade water containing 0.1% formic acid. Mass spectrometry analysis was performed on a linear ion trap LTQ mass spectrometer (Life Technologies, Shanghai, China) as per manufacturer’s guidelines. Of note, the instrument was operated in positive ion mode and MS full scans were recorded over a mass range of 400–1600 m/z. Post-acquisition of data, ReAdW was used to convert files to mzXML, which were used to query the human IPI database using the Sequest platform. False discovery rate was determined using the Trans-Proteomic Pipeline TPP, with a FDR set at approximately 5%. Finally, relative enrichment or depletion in tumor group was normalized to control group.

### Isolation of miRNA, mRNA and qRT-PCR

RNA was isolated using Trizol (Life Technologies, Shanghai, China) as per manufacturer instructions. The expression level of *PCBP1* mRNA and *GAPDH* mRNA were detected by TaqMan miRNA assays (Life Technologies, Shanghai, China). MiRNA from cells and tissues were extracted by the mirVana miRNA isolation kit (ThermoFisher Scientific, Shanghai, China) according to the manufacturer instructions. The expression levels of indicated miRNAs and *RNU6B* were detected by TaqMan miRNA assays (Life Technologies, Shanghai, China). The −ΔΔCt method was used to analyze the data in each case and normalization was done to *GAPDH* and *RNU6B* expression for mRNA and miRNA, respectively.

### RNA immunoprecipitation

Tissue specimens from 5 patients were lysed for 15 minutes on ice in a lysis buffer containing 100 mM KCl, 5 mM MgCl_2_, 10 mM HEPES, pH 7.0, 0.5% NP-40 detergent supplemented with fresh 1 mM DTT, 1000 units/ml RNasin (Promega, Madison, WI, USA), and Mini protease inhibitor cocktail (Roche Diagnostics, Indianapolis, IN, USA). The post-nuclear fraction was collected by spinning the cells at 15,000 *rcf*f for 15 minutes at 4 °C, and 10% (v/v) was removed for input sample. Four milligrams of lysate was utilized for immunoprecipitation using 10 µg of mouse monoclonal anti-PCBP1 antibody (clone E-2, sc-137249; Santa Cruz Biotechnology, USA) or 10 µg of mouse IgG (Santa Cruz Biotechnology, Santa Cruz, CA, USA) using Pierce Crosslink IP Kit (Life Technologies, Shanghai, China). Aliquots were sequestered to confirm successful immunoprecipitation via SDS-PAGE. The rest of the immunoprecipitated complex was digested with 30 μg of proteinase K to release the ribonucleoprotein complex. TRIzol LS reagent (Life Technologies, Shanghai, China) was subsequently used to extract miRNA from the immunoprecipitates and the input samples following manufacturer’s recommendations. The miRNAs isolated above were templated for qRT-PCR as described above. Fold enrichment above the sample specific background (linear conversion of the first ΔΔCt) was calculated using the formula: Fold Enrichment = 2^(−ΔΔCt [RIP/NS])^. Data are presented as mean ± SD.

### Immunohistochemistry

Tissue sections (5 μm) were de-paraffinized and rehydrated through a graded alcohol series. Antigen retrieval was performed using 1X Target Retrieval Solution (Dako, Carpinteria, CA, USA). Endogenous peroxidase was blocked using 3% hydrogen peroxide for 10 minutes. Sections were incubated with primary antibody against PCBP1 (ab133421; Abcam, Waltham, MA, USA) (1:250 dilution) at 4 °C for overnight. Following wash and incubation with anti-goat secondary antibody at room temperature for 1 hour, immunoreactivity was detected using diaminobenzidine (Innovex Biosciences, Richmond, CA, USA) as a chromogen. Slides were counter-stained with hematoxylin before imaged with microscope at different magnifications.

### Cell culture and treatment

MKN45 cell lines were purchased from the cell bank of Chinese Academy of Sciences. Cells were maintained in RPMI1640 medium (Life Technologies, Shanghai, China) supplemented with 20% FBS (Lonza, Germany). All cells were cultured in a 5% CO_2_ humidified atmosphere and at 37^0^ C. Where indicated cells were treated with indicated dose of Docetaxel.

### Plasmids and transfection

*PCBP1* plasmid was obtained from Open Biosystems. The miR-3978 mimic was bought from ThermoFisher Scientific. Where indicated cells were either transfected with *PCBP1* overexpression construct or the miR-3978 mimic or miR-6124 mimic for 12 hours using Lipofectamine 3000 (ThermoFisher Scientific, Shanghai, China). In indicated cases, cells were transfected with both miR-3978 mimic and either siRNA targeting Luciferase or *PCBP1* (Sigma-Aldrich, Shanghai, China).

### MTT assay

The MTT assay kit (Sigma-Aldrich, Shanghai, China) was used to measure cell proliferation rates. The relative optical density (OD) was measured at 570 nm and data is represented as mean ± standard deviation from at least three independent experiments.

### Statistical analyses

All analyses were done by using the SPSS statistical software program version 18.0 (IBM Corporation, NY, USA). P-values < 0.05 were considered statistically significant.

## Results

**Figure 1:**
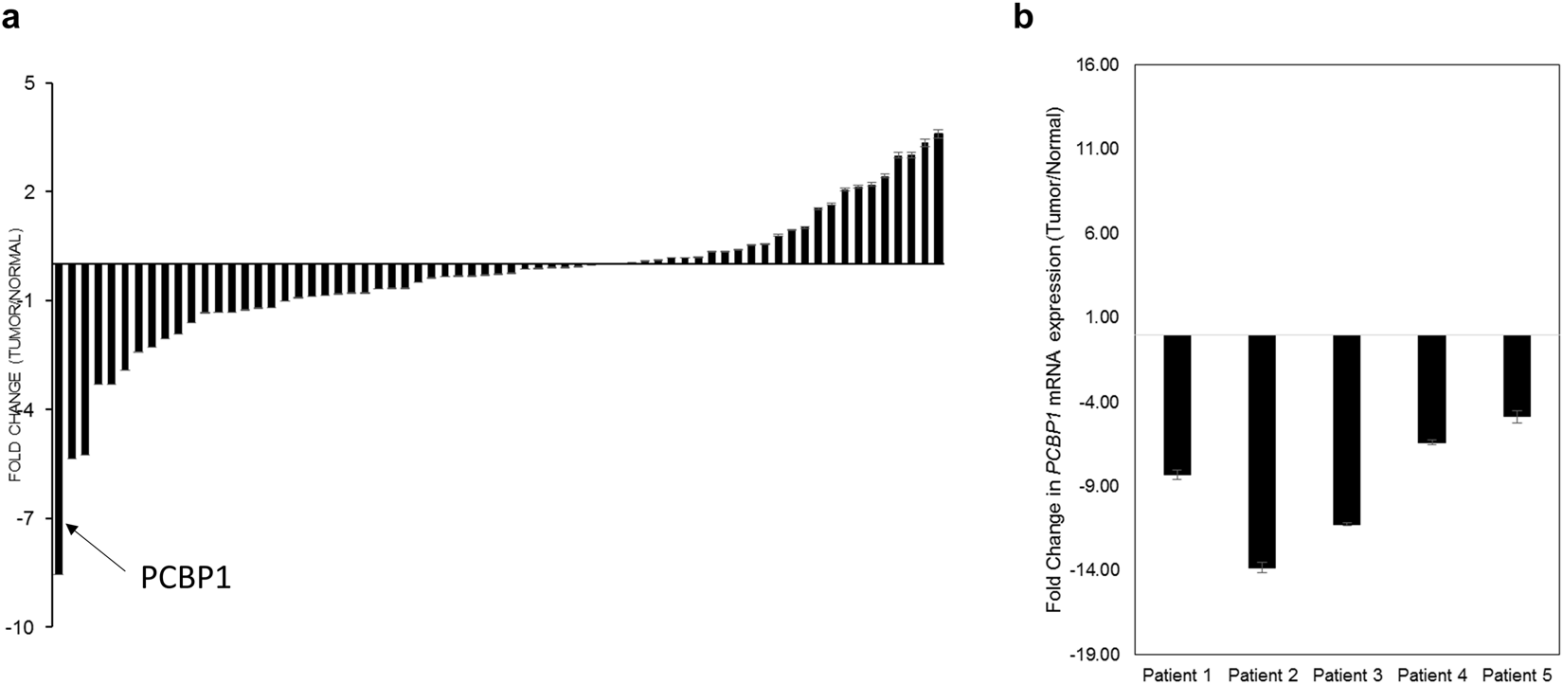
PCPB1 is differentially expressed in gastric cancer patients with peritoneal metastasis. (**a**) Differential protein expression determined by mass spectrometry in metastatic versus adjacent control tissue specimens (n = 5). Shown are log_2_ ratio of expression of normalized values compared to the control group. Data are mean + standard deviation of 5 pairs of patient specimens. (**b**) Evaluation of *PCBP1* mRNA expression in the five patients groups. Data was normalized to *GAPDH* expression and expressed as compared to the corresponding control group. Data are mean ± standard deviation of three independent replicates.

Given that miR-3978 expression is downregulated during peritoneal metastasis, we wanted to determine the underlying mechanism of altered miR-3978 expression. Mass spectrometry analysis of protein expression from tissues obtained from patients with metastatic gastric cancer and tumor adjacent normal tissue revealed that whereas five proteins (PCBP1, DSP, FZD7, ITGA5, and MMP2) were downregulated at least 3 folds (log2), four proteins (IGFBP4, KRT19, NODAL, and PRP4A1) were upregulated at least 3 folds (log2) in metastatic tumor tissue specimens compared to control tissue specimens. Poly r(C) binding protein (PCBP1), also known as heterogeneous nuclear ribonucleoprotein E1 (hnRNP E1), was the most downregulated protein in the metastatic samples (−8.54 ± 1.12 folds) (Fig. 1a). Given that PCBP1 has been indicated in the pathogenesis of a wide variety of cancers^28–34^, we decided to further pursue the role of PCBP1 in peritoneal metastasis of gastric cancer. Since, PCBP1’s tumor suppressor role can be attenuated either by its loss of expression or Akt2-mediated phosphorylation^29^, we next evaluated expression of *PCBP1* messenger RNA (mRNA) in metastatic gastric cancer patient samples. As shown in Fig. 1b, *PCBP1* mRNA was downregulated 12.2 ± 1.55 folds in metastatic tissue compared with normal peritoneum, indicating that loss of *PCBP1* expression might be the pervasive mechanism dictating its attenuation of tumor suppressor role in the context of peritoneal metastasis in gastric cancer.

**Figure 2:**
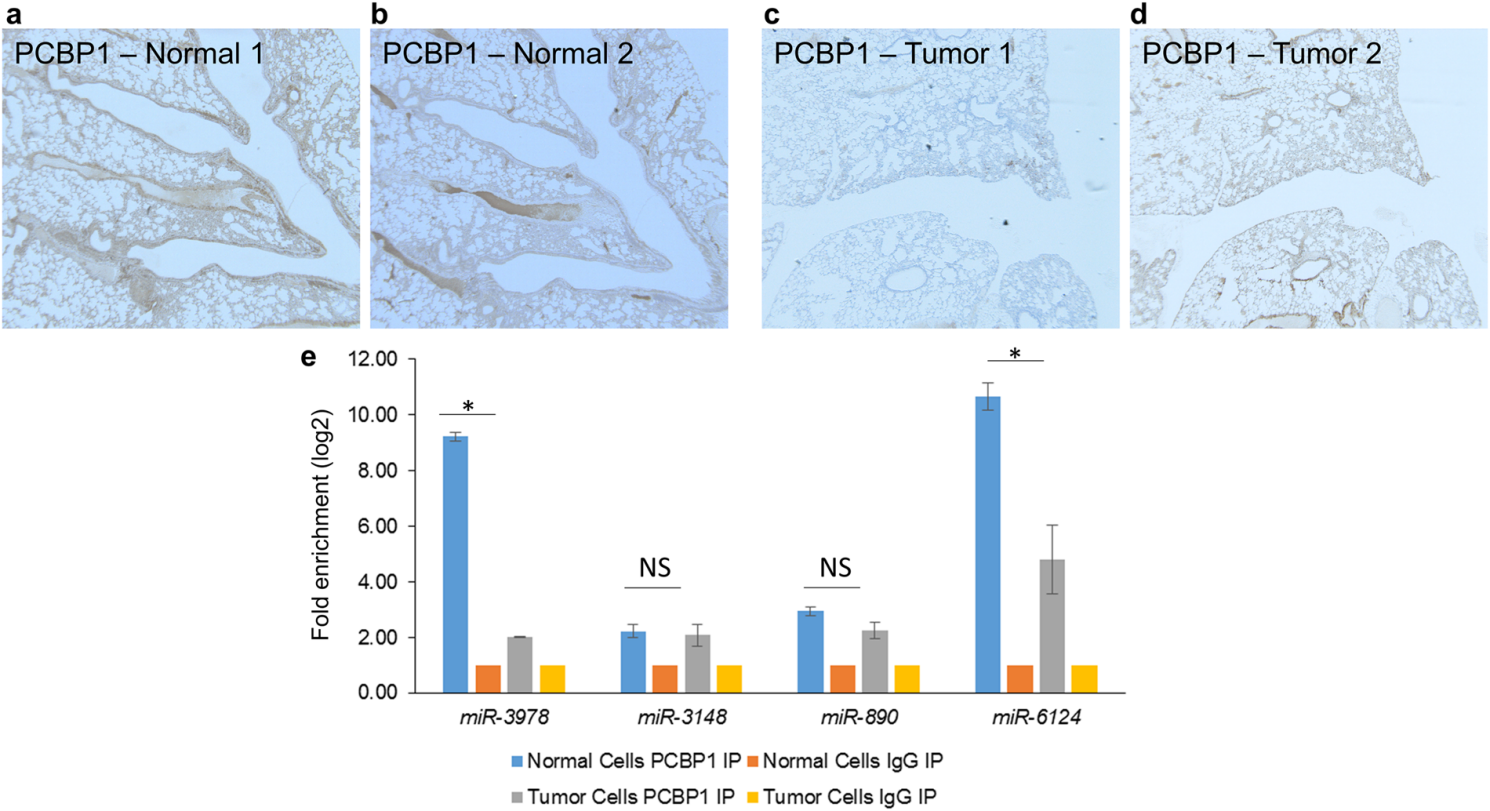
PCBP1 immunoprecipitates from gastric cancer patients with peritoneal metastasis are enriched in miR-3978. (**a**–*d*) Immunohistochemical staining of PCBP1 expression in representative normal (**a**,**b**) and tumor tissue sections (**c**,**d**). Scale bar, 100 µm. (**e**) Shown are relative enrichment of indicated miRNA in PCBP1 or mouse IgG immunoprecipitates as determined by qRT-PCR of lysates made from metastatic and normal adjacent tissue specimens. Data shown are mean ± standard deviation of three independent replicates.

Immunohistochemical staining of PCBP1 in representative tissue specimens obtained from metastatic tissue or tumor adjacent normal tissue exhibited robust PCBP1 staining in the control peritoneal tissues, which was significantly attenuated in the tumor tissues (Fig. 2a–d). PCBP1 staining in metastatic tumor tissue was observed at comparatively much less intensity (Fig. 2c,d), confirming *in vivo* loss of PCBP1 expression might play a role in promoting peritoneal metastasis.

To determine if altered *PCBP1* expression was a coincidental occurrence or was also correlated to differential miR-3978 expression in these patients, we performed RNA immunoprecipitation using anti-PCBP1 antibody. Immunoprecipitation with anti-PCBP1 antibody, but not a control mouse IgG, showed significant enrichment of miR-3978 and miR-6124, but not miR-3148 or miR-390 in normal tissue compared to tumor tissue (Fig. 2e). Each of the miRNA tested for enrichment were predicted to be putative ones regulating legumain expression^17^. Cumulatively, this suggested that PCBP1 expression levels (high in normal and low in peritoneal metastatic tissues) correlate directly with miR-3978 expression levels in these tissues. Our results are indicative of a potential common regulatory mechanism underlying *PCBP1* and miRN-3978 expression.

**Figure 3:**
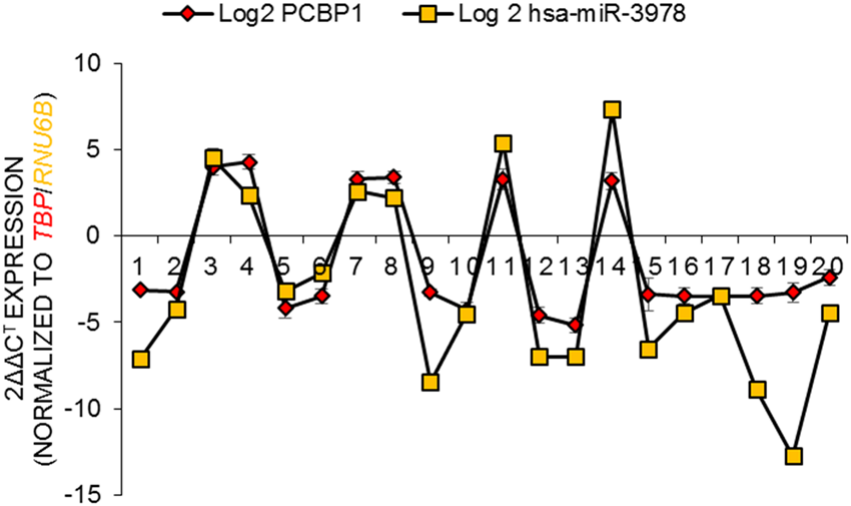
MiR-3978 and PCBP1 levels are directly correlated in gastric cancer patients. MiR-3978 and *PCBP1* mRNA expression was determined in 20 patients by qRT-PCR. Data shown are mean ± standard deviation of three independent replicates.

Given that our experiments indicated that miR-3978 and *PCBP1* expression are correlated, we hypothesized that suppression of *PCBP1* expression might be an underlying feature of peritoneal metastasis pathogenesis, much like what is observed for miR-3978^17^. We determined miR-3978 and *PCBP1* expression in 20 patients. Our results indicated a dynamic and direct correlation between expression of miR-3978 and *PCBP1* (Fig. 3) (P < 0.005, Pearson correlation r = 0.9361).

**Figure 4:**
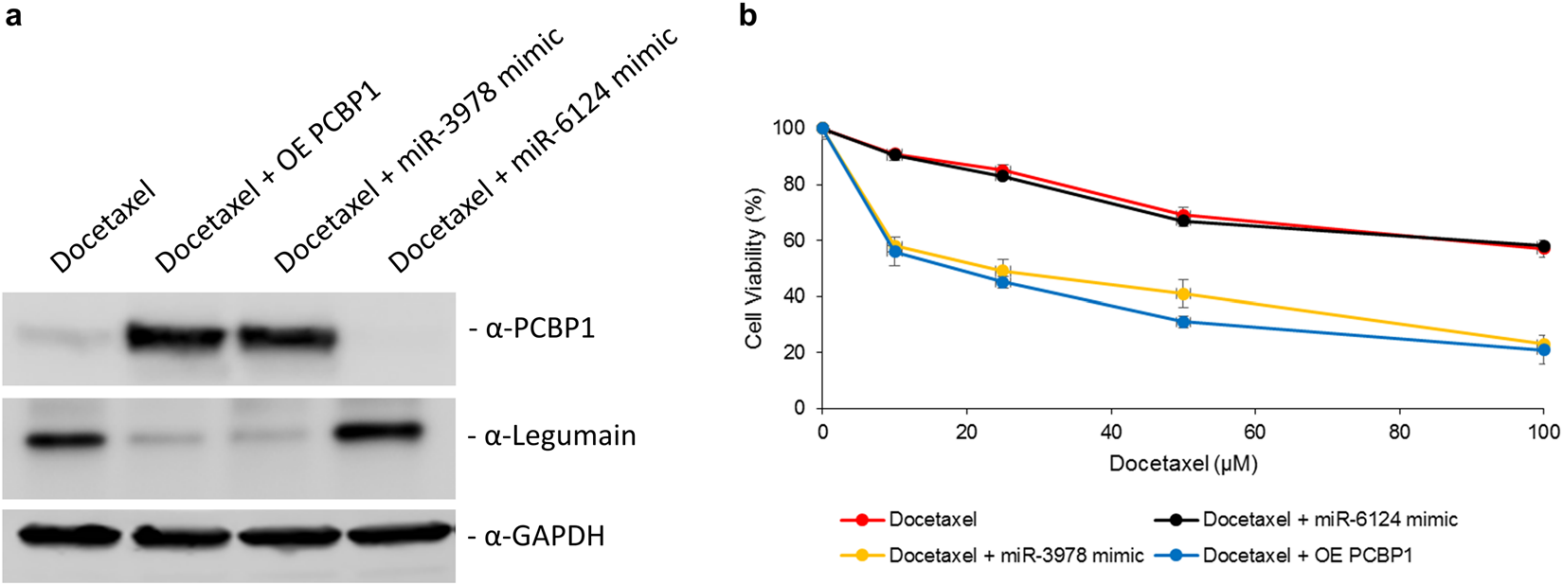
Ectopic expression of either miR-3978 or *PCBP1* levels increased sensitivity to docetaxel. MKN45 cells were either not transfected or transiently transfected with either miR-3978 mimic, miR-6124 mimic, or *PCBP1* for 12 hours. The cells were then treated with indicated doses of docetaxel for 72 hours. Post-treatment the cells were either processed for western blot or MTT assay was used to determine cell viability. (**a**) Immunoblot analysis of PCBP1 and legumain expression and (**b**) proliferation under the aforementioned conditions are shown. Data in (**b**) represent mean ± SD of three independent replicates. *Please refer to Supplementary* Figure 1a,b *for full-length blots shown in (****a****)*. *Please note PCBP1 and GAPDH were from the same gel*, *whereas Legumain was from a different gel*. *Samples ran were from experiments processed at the same time*.

We next determined if rescue of miR-3978 and *PCBP1* expression can function as chemosensitizer. We evaluated the effect of miR-3978 mimic-mediated and ectopic *PCBP1* overexpression-mediated replenishment on the cytotoxicity of docetaxel on the well-differentiated cell line mimicking peritoneal metastasis, MKN45 cells. Interestingly, miR-3978 mimic, but not miR-6124 mimic, induced PCBP1 protein expression in the MKN45 cells (Fig. 4a). Legumain expression was suppressed following miR-3978 mimic transfection. Transfection of miR-3978 mimic, but not miR-6124 mimic, significantly chemosensitized MKN45 cells to docetaxel treatment (p < 0.05) (Fig. 4b). Similarly overexpression of *PCBP1* also suppressed legumain expression in MKN45 cells and significantly increased chemosensitivity of the cells to docetaxel treatment (p < 0.05) (Fig. 4b). Cumulatively, these results indicate that miR-3978 or *PCBP1* replenishment sensitizes peritoneal metastatic cells to the cytotoxicity of docetaxel.

**Figure 5:**
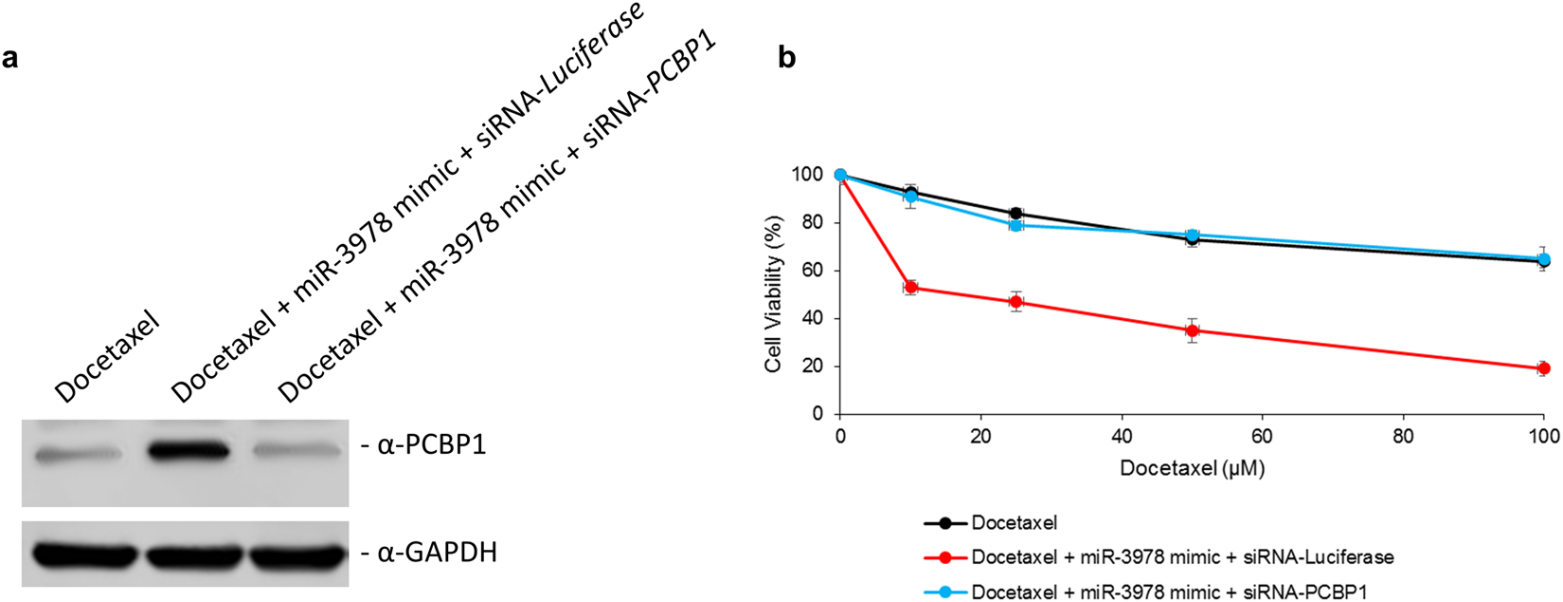
Both miR-3978 and *PCBP1* expression is required to potentiate sensitivity to docetaxel. MKN45 cells were either not transfected or transiently co-transfected with either miR-3978 mimic and *Luciferase* siRNA, or miR-3978 mimic and *PCBP1* siRNA for 12 hours. The cells were then treated with indicated doses of docetaxel for 72 hours. Post-treatment the cells were either processed for western blot or MTT assay was used to determine cell viability. (**a**) Immunoblot analysis of PCBP1 expression and (**b**) proliferation under the aforementioned conditions are shown. Data in (**b**) represent mean ± SD of three independent replicates. *Please refer to Supplementary* Figure 2a,b *for full-length blots shown in (****a****)*.

To determine if miR-3978 and PCBP1 interaction was important for their individual potency of sensitizing cells to docetaxel treatment, MKN45 cells were transfected with miR-3978 mimic and either a siRNA targeting *Luciferase* or *PCBP1*. Similar to what was observed in Fig. 4a, miR-3978 mimic induced PCBP1 protein expression in the MKN45 cells, which was prevented in cells transfected with siRNA-targeting *PCBP1* (Fig. 5a). Transfection of miR-3978 mimic, significantly chemosensitized MKN45 cells to docetaxel treatment (p < 0.05), which was completely attenuated once *PCBP1* was suppressed in these cells (Fig. 5b). Our results thus indicate that interaction or presence of both PCBP1 and miR-3978 is essential to potentiate chemosensitivity to docetaxel treatment in gastric cancer patients with peritoneal metastasis.

## Discussion

In the current study, our experimental results show that miRNA-3978 and *PCBP1* is downregulated in gastric cancer patients with peritoneal metastasis. MiR-3978 seems to be functioning as part of PCBP1-mediated ribonucleoprotein complexes in the normal peritoneum and such enriched complexes are absent in metastatic tissue.

There are two possibilities – miR-3978 and *PCBP1* are dictating each other’s expression, or they are regulating each other’s function. In the current study, we observed that in metastatic tissue, miR-3978 enrichment in *PCBP1* immunoprecipitates are significantly downregulated. This can obviously stem from the downregulation of *PCBP1* in the metastatic tissues. However, we have earlier shown that miR-3978 expression is also suppressed during metastatic progression. So are these related? Replenishment of miR-3978 upregulated PCBP1 protein expression in the MKN45 cells (Fig. 4a), but ectopic overexpression of *PCBP1* did not increase miR-3978 expression (*data not shown*). Hence, at least in this context it seems that miR-3978 can regulate expression of *PCBP1*. However, miRNA prediction algorithms did not predict putative binding site of miR-3978 in the 3′UTR of *PCBP1* mRNA (*data not shown*). This will indicate that miR-3978 is suppressing a negative regulator of *PCBP1*, and hence indirectly mimicking *PCBP1* expression patterns. Such an observation is not without precedence. In fact, it has also been shown that miRNAs and their associated ribonucleoprotein complexes are amenable to various modulations that directly affect both their expression and function^35,36^.

The RNA binding protein, PCBP1 is well characterized for its role as a tumor suppressor. It has been shown that either loss of PCBP1 expression or phosphorylation at serine 43-mediated post translational modification has a pro-tumorigenic and pro-metastatic effect in pancreatic, ovarian, breast, lung, gastric, and Burkitt lymphoma^28–34^. The paralog of PCBP1, PCBP2, has been shown to regulate the miRNA biogenesis pathway based on cytosolic iron levels^37^. It was shown that PCBP2 associates with Dicer and thus regulates processing of miRNA precursors and that cytosolic iron levels determined whether PCBP2 could physically interact with Dicer and to the miRNA precursors^37^. Our experiments showed a direct interaction of PCBP1 and miR-3978 that are repressed during peritoneal metastasis of gastric cancer. It will be important to determine if PCBP1 dictates this miRNA modulation through a similar mechanism by interacting with Dicer and potentiating miRNA processing from the precursor pre-miRNAs. At least in the context of cellular iron homeostasis, PCBP1 was shown not to interact with Dicer^37^. If a similar scenario is observed in gastric cancer, it will have to be determined what alternate nodes in the miRNA biogenesis pathway are being modulated by differential PCBP1 expression, and *vice versa*.

Defining the mechanism(s) that regulates miR-3978 and PCBP1 expression and deciphering whether they are in the same or different regulatory lineages will be important. PCBPs are known to act as transcriptional regulators by binding to *cis* elements in gene promoters which in turn interact with RNA polymerase II transcription machinery^38^. This will explain widespread effect of suppressed PCBP1 mRNA expression on RNA polymerase II function. The PCBPs itself is also regulated by their cellular localization. It has been shown that p21-activated kinase 1 (PAK1) increases promoter activity of PCBP1^38^. PAK1 and Akt2 both lie downstream of signaling pathways activated during metastatic progression, like TGF-beta signaling pathway. Whereas Akt2-mediated phosphorylation of PCBP1 has been shown to inhibit its RNA-binding capacity (resulting in translational activation of EMT inducer genes)^29^, PAK1 inactivation can lead to transcriptional downregulation of PCBP1^38^. It will be interesting to determine if miR-3978 and PCBP1 share similar promoter architecture and whether they are both regulated by PAK1 in the context of peritoneal metastasis in gastric cancer.

Cumulatively, our findings highlight both loss of miR-3978 expression and *PCBP1* as potential prognostic biomarker in gastric cancer patients. It will be interesting to investigate in future research endeavors how miR-3978, *PCBP1*, and legumain expression varies and correlates to disease progression in patients that had either radiation therapy or adjuvant chemotherapy post-radical resection of the primary tumor.

## Electronic supplementary material

Supplementary Information
