# Author Correction: Expression of both poly r(C) binding protein 1 (PCBP1) and miRNA-3978 is suppressed in peritoneal gastric cancer metastasis

**DOI:** 10.1038/s41598-018-26795-6

**Published:** 2018-06-01

**Authors:** Fu-jian Ji, Yuan-yu Wu, Zhe An, Xue-song Liu, Jun-nan Jiang, Fang-fang Chen, Xue-dong Fang

**Affiliations:** 1 Department of Gastrointestinal Colorectal and Anal Surgery, China-Japan Union Hospital of Jilin University, Changchun, 130033 China; 2 Department of Cardiology, China-Japan Union Hospital of Jilin University, Changchun, 130033 China

Correction to: *Scientific Reports* 10.1038/s41598-017-15448-9, published online 14 November 2017

The original version of this Article contained incorrect email addresses for Fang-fang Chen and Xue-dong Fang, which were incorrectly listed as xue-198627@163.com and fangxuedong_111@163.com respectively. This error has now been corrected in the HTML and PDF versions of the Article.

