# Supplementary material for: Expression of both poly r(C) binding protein 1 (PCBP1) and miRNA-3978 is suppressed in peritoneal gastric cancer metastasis
Source: Sci Rep. 2017 Nov 14;7:15488. doi: 10.1038/s41598-017-15448-9 (PMC5686074; doi:10.1038/s41598-017-15448-9)

Expression of both poly r(C) binding protein 1 (PCBP1) and miRNA-3978 is suppressed in peritoneal gastric cancer metastasis

Fu-jian Ji, Yuan-yu Wu, Zhe An, Xue-song Liu, Jun-nan Jiang,Fang-fang Chen, Xue-dong Fang

Supplementary Information: Contains full-length blots for western blot images shown in Figures 4

Supplementary Figure 1a – related to Figure 4a. Black dotted box represents portion of gel used for image shown in Figure 4a.

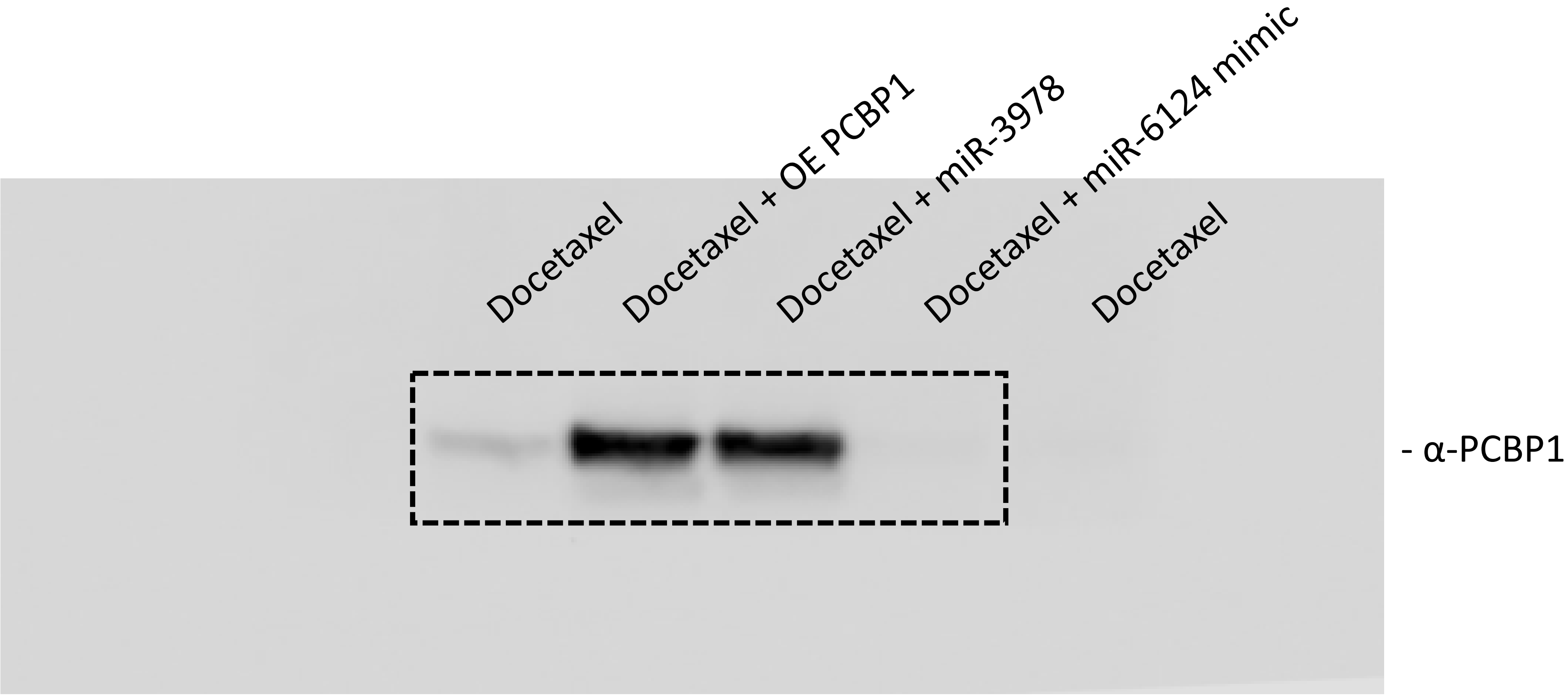

Supplementary Figure 1b – related to Figure 4a. Black dotted box represents portion of gel used for image shown in Figure 4a.

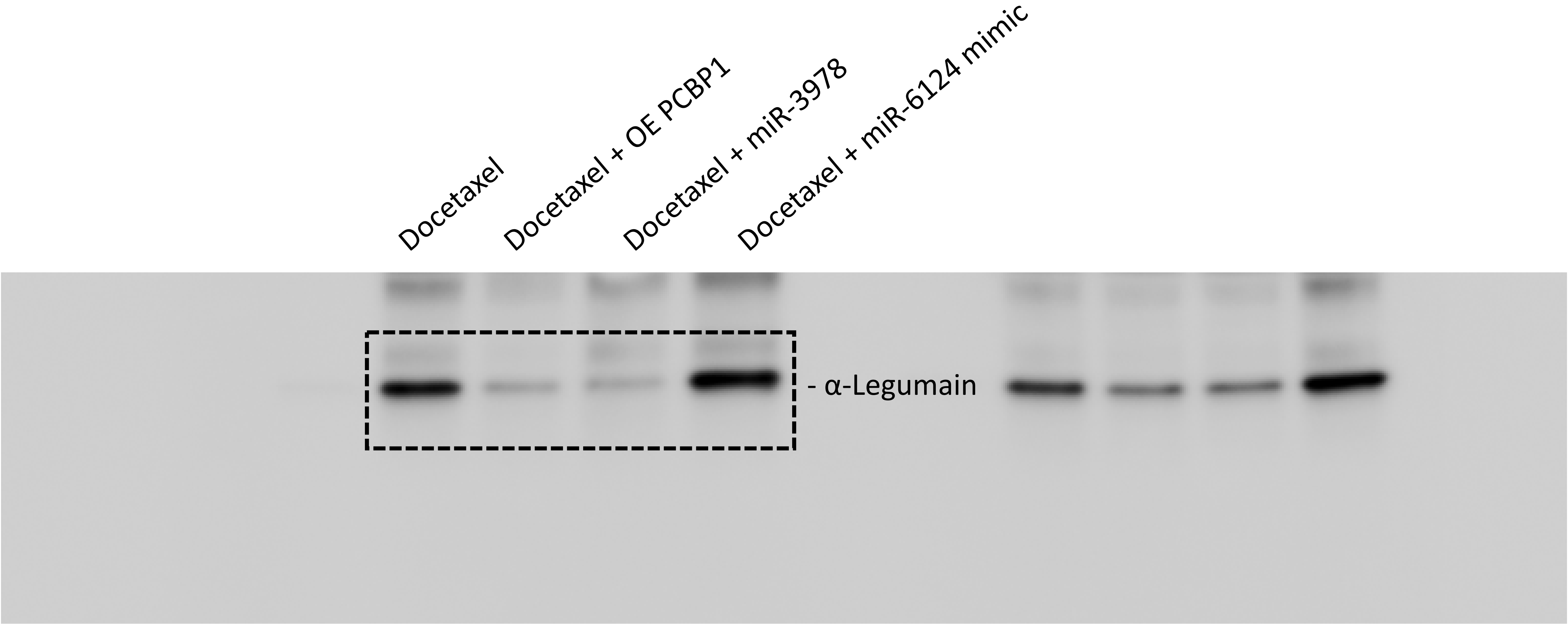

Supplementary Figure 1c – related to Figure 4a. Black dotted box represents portion of gel used for image shown in Figure 4a.

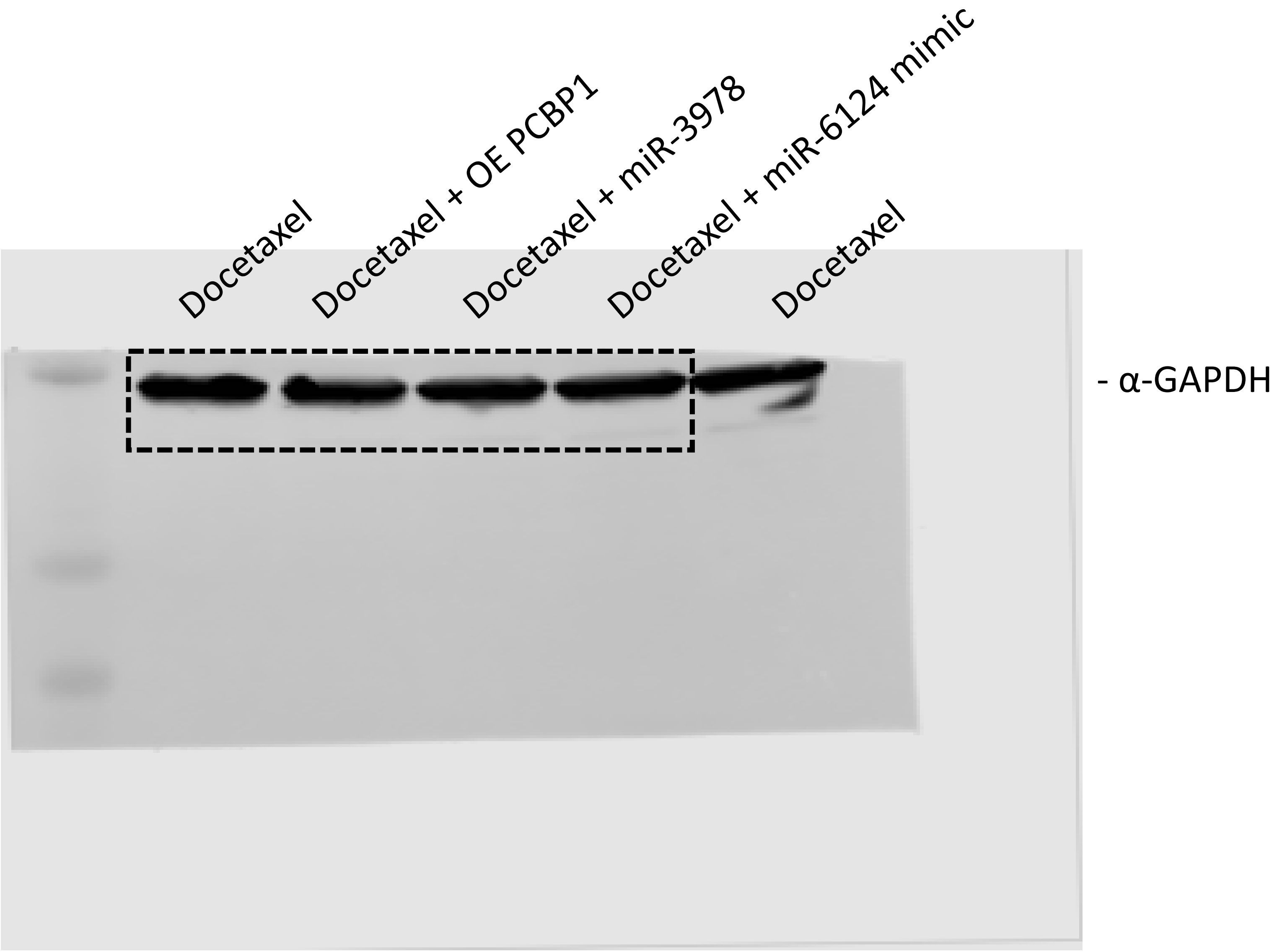

Supplementary Figure 2a – related to Figure 5a. Black dotted box represents portion of gel used for image shown in Figure 5a.

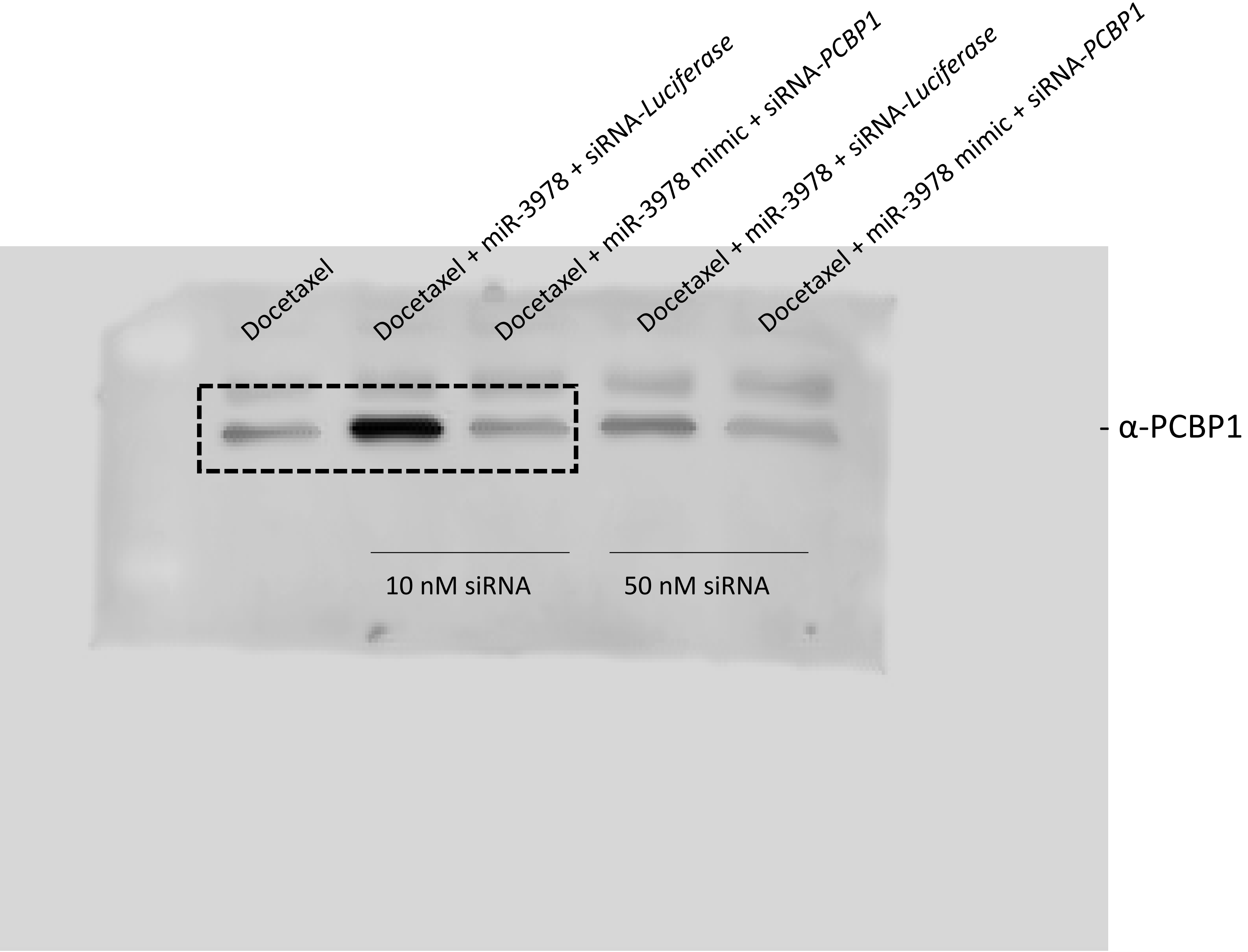

Supplementary Figure 2b – related to Figure 5a. Black dotted box represents portion of gel used for image shown in Figure 5a.

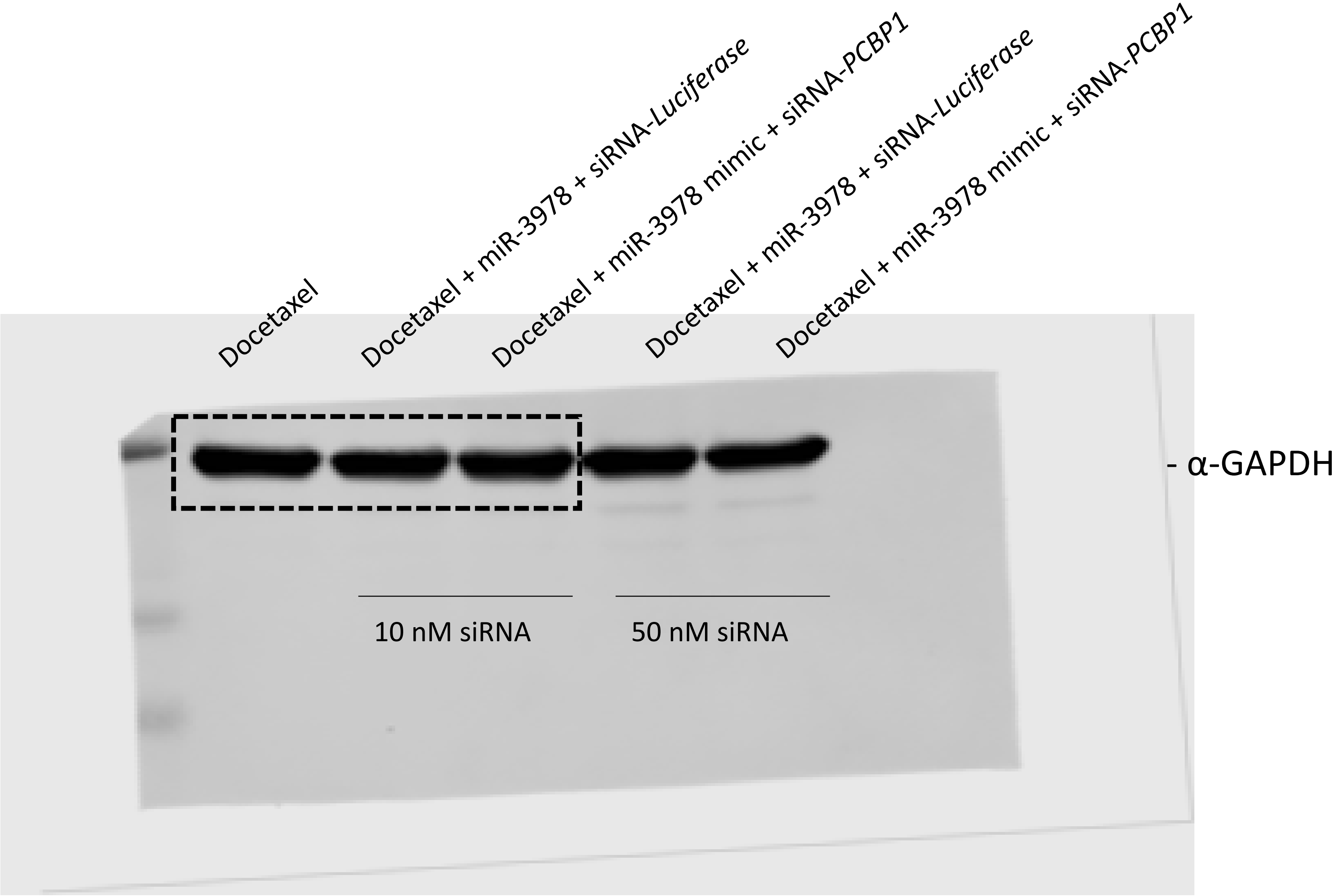

Supplement: Supplementary file 1 — Supplementary Information [file 41598_2017_15448_MOESM1_ESM.pdf]
